# Adsorption characteristics of nickel (II) from aqueous solutions by Zeolite Scony Mobile-5 (ZSM-5) incorporated in sodium alginate beads

**DOI:** 10.1038/s41598-023-45901-x

**Published:** 2023-11-10

**Authors:** Mohamed S. Hellal, Ahmed M. Rashad, Kishore K. Kadimpati, Sayed K. Attia, Mariam E. Fawzy

**Affiliations:** 1 Water Pollution Research Department, National Research Centre, El Behooth St., P.O. Box 12622, Dokki, Cairo Egypt; 2 Analysis and Evaluation Department, Egyptian Petroleum Research Institute, Nasr City, Cairo Egypt; 3 Department of Environmental Biotechnology, Faculty of Energy and Environmental Engineering, Silesian University of Technology, ul. Akademicka 2, 44-100 Gliwice, Poland

**Keywords:** Environmental chemistry, Environmental impact, Nanoparticles, Metamaterials

## Abstract

Nickel, a prevalent metal in the ecosystem, is released into the environment through various anthropogenic activities, leading to adverse effects. This research explored utilizing zeolite scony mobile-5 (ZSM-5) nanoparticles encapsulated in sodium alginate (SA) for nickel (II) removal from aqueous solutions. The adsorption characteristics of SA/ZSM-5 were examined concerning contact duration, initial metal ion concentration, pH level, temperature, and sorbent dosage. The findings revealed that a rising pH reduced Ni (II) uptake by the sorbent while increasing the Ni (II) concentration from 25 to 100 mg L^−1^ led to a decrease in removal percentage from 91 to 80% under optimal conditions. Furthermore, as sorbent dosage increased from 4 to 16 g L^−1^, uptake capacity declined from 9.972 to 1.55 mg g^−1^. Concurrently, SA/ZSM-5 beads' Ni (II) sorption capacity decreased from 96.12 to 59.14% with a temperature increase ranging from 25 to 55 °C. The Ni (II) sorption data on SA/ZSM-5 beads are aptly represented by Langmuir and Freundlich equilibrium isotherm models. Moreover, a second-order kinetic model characterizes the adsorption kinetics of Ni (II) on the SA/ZSM-5 beads. A negative free energy change (ΔG°) demonstrates that the process is both viable and spontaneous. The negative enthalpy values indicate an exothermic nature at the solid–liquid interface while negative entropy values suggest a decrease in randomness. In conclusion, this novel adsorbent exhibits promise for removing nickel from aqueous solutions and could potentially be employed in small-scale industries under similar conditions.

## Introduction

The greatest challenges for decontamination of the environment from hazardous pollutants especially heavy metals are faced by the global community^1,2^. The accumulation of heavy metals has a crucial damage to human health and the ecological environment. Therefore, the release of harmful materials into the water must be planned to sustain a clean environment and prevent hazards. The recent development of human activities and industrialization leads to many environmental problems, especially with water scarcity^3,4^.

The paucity of naturally occurring water supplies has increased pressure on countries to implement zero discharge laws for industrial and domestic purposes^5^. To better control the quality aspect of water, world nations implemented a local strategy and tailored regulatory frameworks^6^. Nowadays, Egypt's water strategy, which strives to effectively manage limited water resources while preserving groundwater and surface supremacy, has recently received a lot of attention^7^. Waste degradation and rainfalls provided a pathway for the penetration of associated heavy metal ions from industrial, agricultural, and household activities into groundwater, which had opposing effects on humans and the surrounding ecosystem in a short and/or long time^8^. Organic compounds, organic halogenated compounds, heavy metals, and fertilizers are the most difficult pollutants that can be removed due to their low biodegradability and complex structure^9^. Heavy metals are produced in the environment from cosmetics, paints, chemicals, electroplating, automobile, dyestuff, and plastics industries^2,10^.

Nickel (II) is one of toxic heavy metals that can be discharged into the environment through industrial wastewater. Nickel is a silvery-white lustrous metal that is involved in several industrial processes such as electroplating, silver refineries, electronics, coins, zinc base casting, stainless steel fabrication, and storage battery industries^11^. The annual amount of Ni released into the environment is estimated to be between 150,000 and 180,000 tons^12^. Chemical reduction, flocculation, filtration, chemical precipitation, evaporation, solvent extraction, biosorption, ion exchange, reverse osmosis, activated carbon adsorption, electrodialysis, electrocoagulation and membrane separation techniques are all common ways for removing Ni (II) from wastewaters^13^. Johnson et al.^14^, studied the removal of Ni(II) from wastewater using coagulation and flocculation enhanced treatment process. They concluded that dosing of 40 mg L^−1^ ferric chloride and 0.5 mg L^−1^ polymer resulted in only 17% removal of Ni (II). Mansoorian et al.^15^, studied the practical application of electrocoagulation processes (ECs) to remove nickel from water solutions using iron rod electrodes. They concluded that 99% of Nickel could be removed from wastewater at contact time of 40 min. Also, Barhoumi et al.^16^, reported a 95% removal of Ni (II) from wastewater using rectangular aluminum electrodes in electrocoagulation process. The use of adsorption process for Ni (II) removal has garnered significant attention as a prevalent method due to its cost-effectiveness, operational simplicity, superior removal efficacy, and exceptional reusability. The critical aspect of the adsorption process is selecting an appropriate adsorbent material. Consequently, researchers are dedicating considerable resources to developing sustainable, economically viable, and minimally toxic adsorbents for enhanced contaminant remediation in aqueous systems^17^. Ni (II) removal by adsorption technique was investigated by Jian et al.^18^ using plant biomass from boiled sunflower head (BSH) and chemically treated sunflower by formaldehyde. They found that utilizing 2.0 g L^−1^ BSH, achieved a percentage of removal 75.9% at pH 6, with a maximum adsorption capacity of 16.26 mg g^−1^. In another study by Jian et al^19^, copper oxide nanoparticles were prepared by the chemical precipitation method and tested for the removal of nickel ions from synthetic water. It was reported that, at ambient temperature, 76.9% of Ni (II) was removed at the optimum conditions after 90 min with a dose of 0.2 g L^−1^ at neutral pH, and the chemosorption process describes the mechanism of adsorption.

Zeolite, a prevalent adsorbent, facilitates the removal of metal ions and cationic dyes from aqueous solutions through adsorption and ion exchange processes, utilizing its unique porous structure^20,21^. It has microporous channels that prevent reactants and products from diffusing, especially bulky molecules. However, the absence of adsorption sites on the surface of zeolite, however, limits its utility in sewage disposal. Surface modification can thus be used to introduce more adsorption sites. Many experiments have been undertaken to circumvent molecular diffusion limitations, such as shrinking zeolite crystal size or producing intra-crystalline microporosity^22^. This can be overcome through the incorporation of nano-particles in gel bead supports that has high adsorption capacity such as sodium alginates (SA), calcium alginate (CA), and polyvinyl alcohol (PVA) because physical and chemical modification of SA can increase its reusability and stability^23^. The most often employed modification procedures to improve the alginate adsorption capacity are graft copolymerization, composite synthesis, and hydrogel bead production^24^. Presently, great efforts have been devoted to the use of nanotechnology in adsorption techniques that have received great attention and are applied in several environmental applications. It has many advantages as it increases the capacity of adsorption, due to the high surface area and special porous structure that make them unique^25^. The incorporation of nano-materials with SA is currently gaining attention for several applications such as biohydrogen production^26^, and ammonia removal from wastewater^23^.

This study describes the creation of a novel adsorbent material for the removal of nickel from wastewater. To this end, Zeolite Scony Mobile-5 (ZSM-5) nanoparticles were prepared and encapsulated within sodium alginate (SA) beads using calcium chloride as a cross-linking agent. The resulting material was utilized in the adsorption process to remove nickel from aqueous solutions. The effects of various parameters such as nickel concentration, beads dose, adsorption duration, and pH were investigated to optimize the adsorption process.

## Results and discussion

### Characterization of SA/ZSM–5

#### Scanning electron microscopy (SEM) and energy dispersive X-ray spectroscopy (EDX)

**Figure 1:**
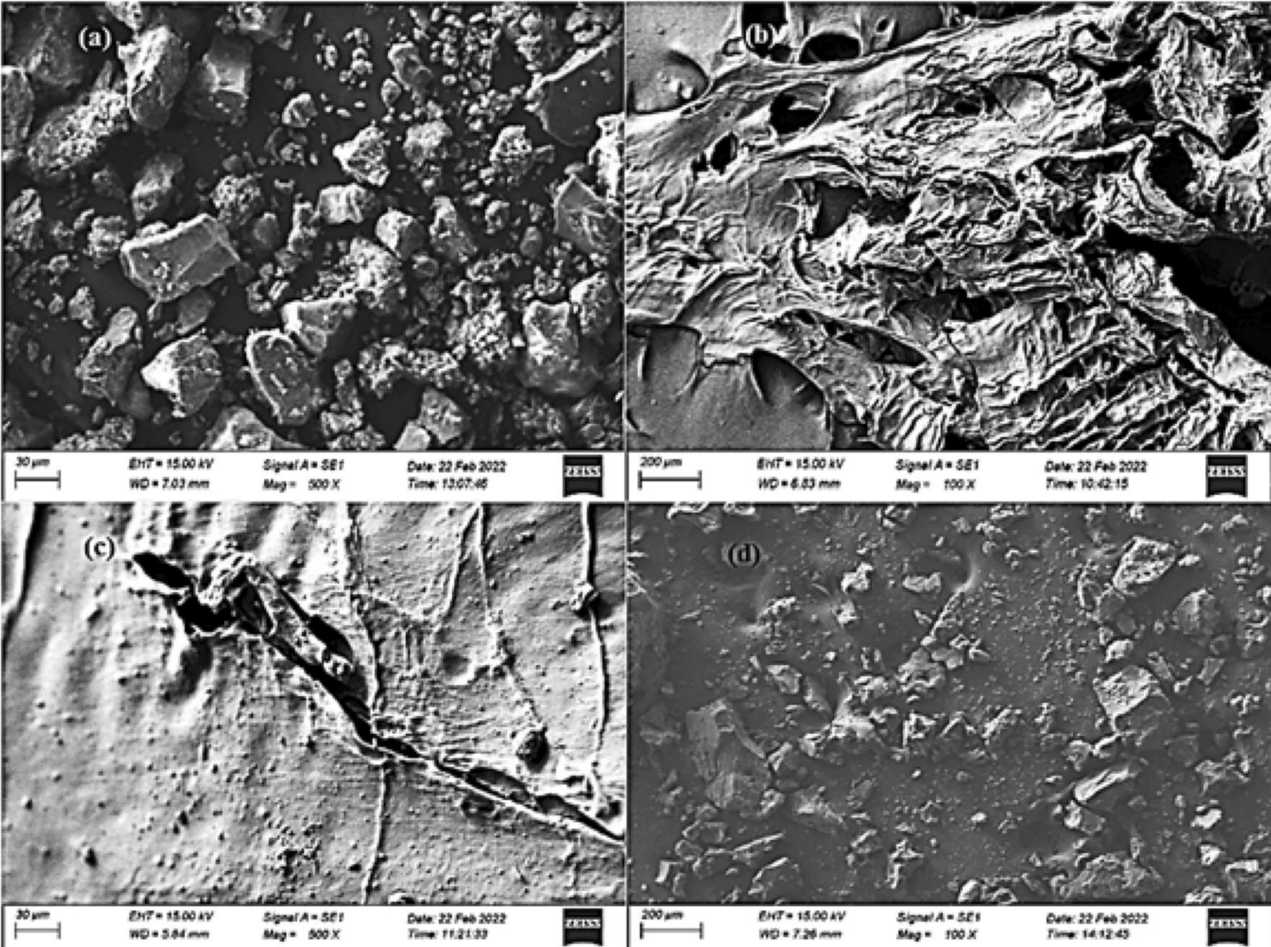
SEM image of ZSM-5 (**a**), SA (**b**), SA/ZSM (**c**), and SA/ZSM loaded with Ni (II) (**d**).

The surface morphology and and elemental composition of SA/ZSM-5 nanospheres were thoroughly examined utilizing Scanning Electron Microscopy (SEM) and Energy Dispersive X-ray spectroscopy (EDX). SEM micrographs portray ZSM-5, SA, and SA/ZSM-5 beads (Fig. 1), highlighting a homogeneous coverage of SA on their external surfaces with no distinct phase of isolated SA particles (Fig. 1c). It is evident that ZSM-5 nanoparticulates predominantly exhibit cuboidal or clustered cuboidal conformations with planar rectangular facets (Fig. 1a). In the case of SA granules, the exterior appeared relatively planar, exhibiting occasional indentations and protrusions. Upon visual inspection, these granules displayed a slightly disordered surface featuring a loose network architecture, heterogeneous pore distribution, and porous structural morphology (Fig. 1b) corresponding to the pre-adsorption state of SA^27^. Composite cross-sectional images revealed a dense ridge-like formation (Fig. 1c), indicative of chemically interlinked porous three-dimensional frameworks ensuing from the amalgamation of SA particulates and ZSM-5. Conversely, the surface texture of SA/ZSM-5 beads displayed rough features accompanied by irregularly sized pores and 'fractures'^28^. Moreover, Ni(II) ions were integrated within the superficial layers of the SA/ZSM–5 beads to generate an encapsulated surface that prevents roughness or crack propagation (Fig. 1d).

**Figure 2:**
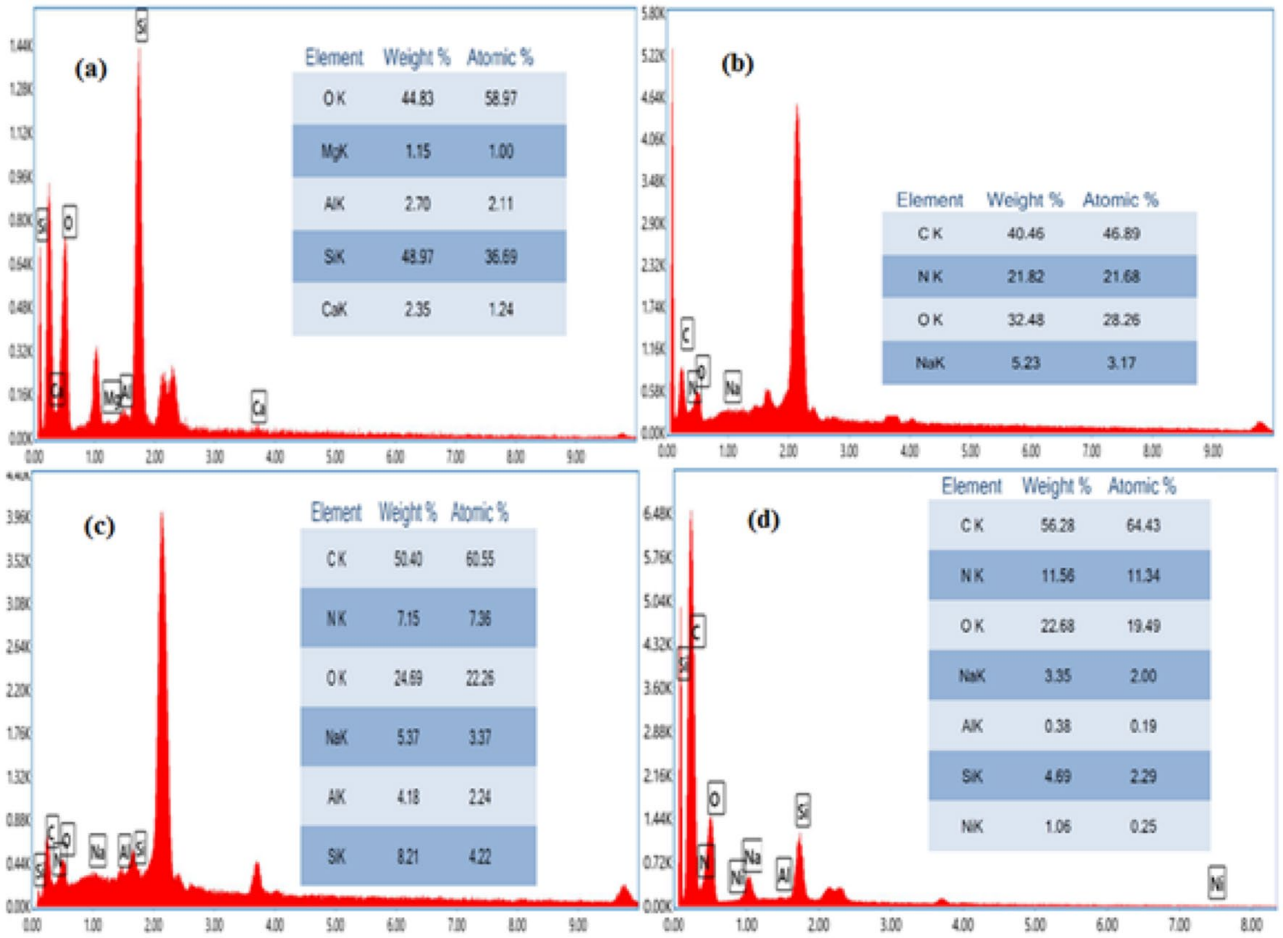
EDX patterns of ZSM-5 (**a**), SA (**b**), SA/ ZSM-5 (**c**), and SA/ZSM-5 loaded with Ni (II) (**d**).

Further insight into the constituent components of SA/ZSM–5 granules pre- and post-Ni(II) adsorption is provided by EDX analyses. As depicted in Fig. 2a–c, not all spectral patterns exhibited Ni prior to adsorption; however, Ni peaks emerged in the EDX spectrum post-adsorption as shown in Fig. 2d. The presence of nickel peaks in the EDX spectrum confirms the uptake of Ni(II) ions by SA/ZSM-5 nanospheres. C, N, O, Na, Al, and Si were distinctly observed in the EDS imagery of the SA/ZSM-5 composite (Fig. 2c), with their respective weight percentages being 60.55%, 7.15%, 24.69%, 5.37%, 4.18%, and 8.21%. The identification of Al, Na, and Si within the SA/ZSM-5 composite verifies the incorporation of ZSM-5. The parent material was encapsulated with SA beads, and observations revealed particle distribution patterns within these beads as well as mesoporous particle aggregation phenomena—most likely attributable to polymer viscosity^29,30^. Furthermore, the detection of sodium (Na) in the EDX spectra of SA/ZSM-5 supports the successful integration of sodium-alginate within the ZSM-5 framework.Further insight into the constituent components of SA/ZSM–5 granules pre- and post-Ni(II) adsorption is provided by EDX analyses.

#### X-ray diffraction (XRD)

**Figure 3:**
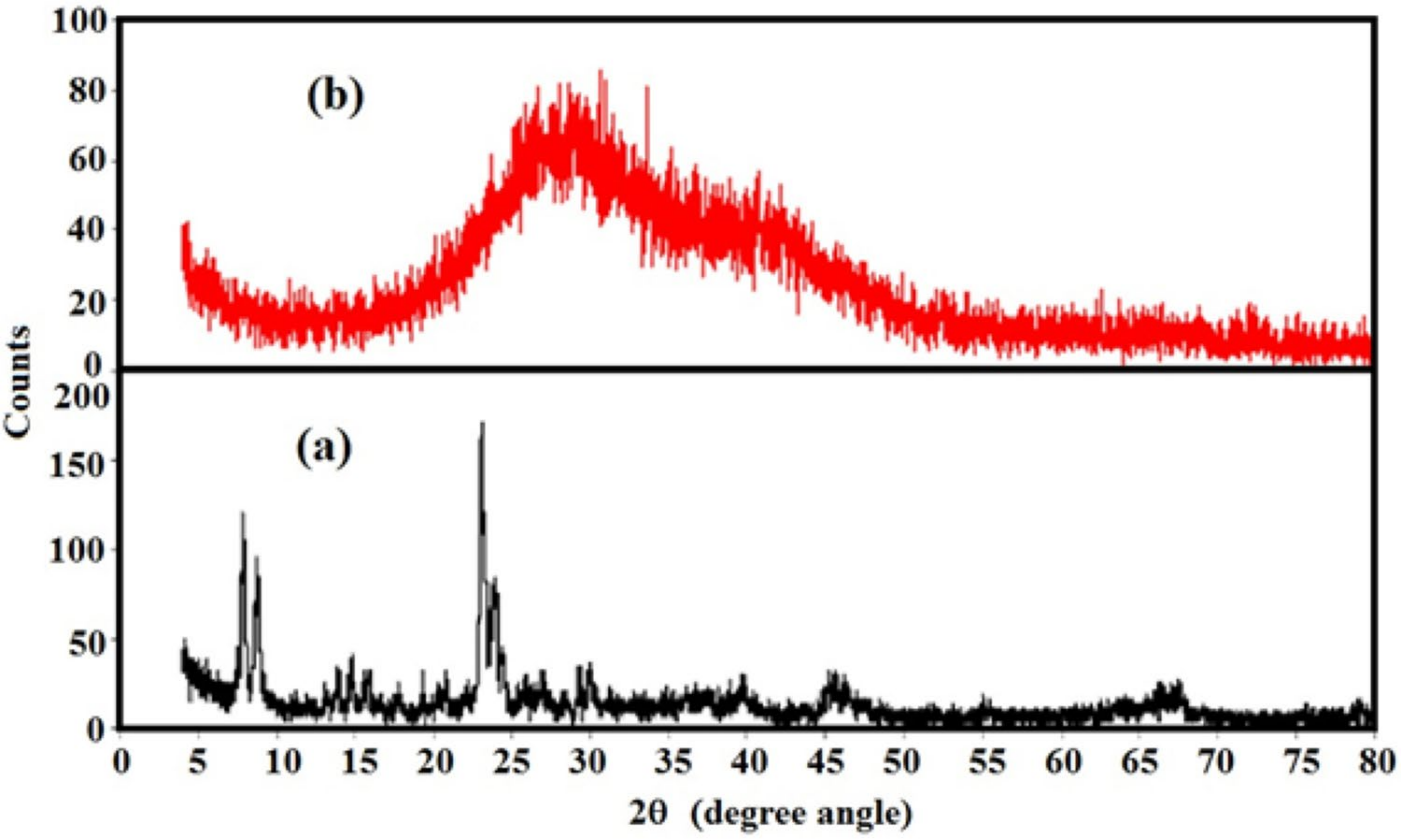
X-RD pattern of, (**a**) ZSM-5, and (**b**) SA/ZSM–5.

X-ray diffraction (XRD) analyses were conducted to evaluate the thermal behavior of both SA and the SA/ZSM-5 composite with respect to crystallinity after undergoing thermal treatment (Fig. 3a, b). The XRD pattern of ZSM-5 exhibited diffraction peaks at 2θ = 7.9°, 8.9°, 23.07°, and 24.01° positions, which correspond to the diffractions of [101], [200], [332], and [051] MFI planes, respectively. This successful crystallization aligns with standard diffraction patterns as reported in previous literature XRD studies were performed to assess the thermal behavior of SA and SA/ZSM-5 composite on crystallinity after thermal treatment (Fig. 3a, b)^31^. The weak, broad peak observed around 27.21° in SA can be attributed to low crystallinity and the presence of an amorphous structural orientation.

In the fabricated nano-composite (SA/ZSM-5), the absence of clear, sharp peaks (Fig. 3b) indicates that nanofillers are uniformly dispersed within the SA matrix and effectively serve as a protective barrier^32^. The disordered, wide-ranging, stunted, and low-intensity peaks signify the amorphous structure shared by both adsorbent materials. Although amorphous materials are macroscopically isotropic with extended-range maxima, their corresponding X-ray patterns often indicate short-range order characteristics, as demonstrated in Fig. 3b. The absence of primary and secondary X-ray reflections from ZSM-5 in SA/ZSM-5 suggests that clay layers in the SA matrix may have exfoliated or that the stacked platelets of ZSM-5 are highly encapsulated and scattered randomly in the SA matrix (Fig. 3b). The lack of primary and secondary X-ray reflections from ZSM-5 in the SA/ZSM-5 composite implies that clay layers within the SA matrix may have experienced exfoliation or that ZSM-5's stacked platelets are highly encapsulated and dispersed randomly throughout the SA matrix (Fig. 3b).

#### Thermogravimetric analysis (TGA)

**Figure 4:**
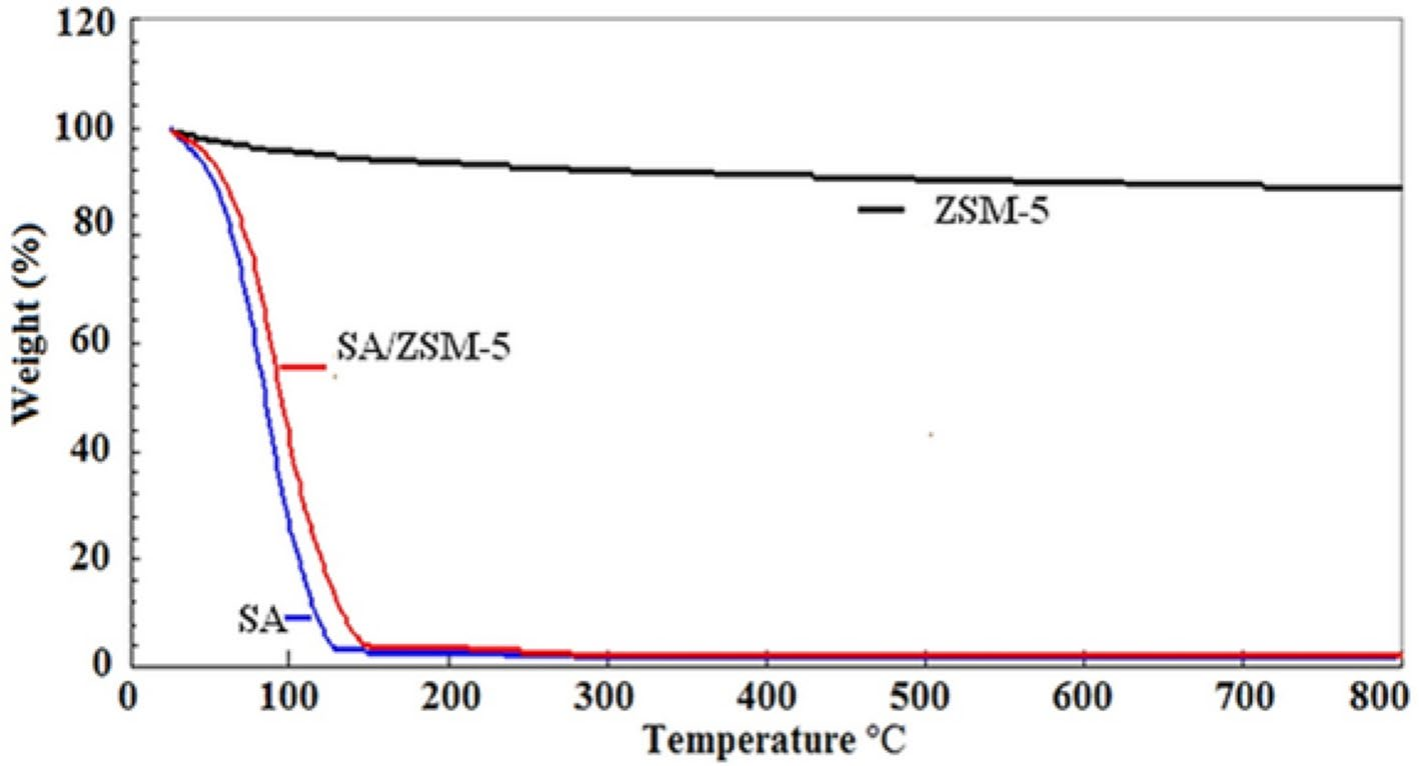
Thermogravimetric analysis for the assessment of thermal behavior of ZSM-5, SA/ZSM-5, and alginate.

ZSM–5 exhibited two thermal stages between 42 and 150 °C and mass loss of 5.88% was noted due to the loss of physiosorbed water and interlayer water (Fig. 4). Three basic thermal events were observed by the SA TGA curve. The first phase, which took place between 30 and 150 °C, is related to the physical absorption of water. The sodium carbonate (Na_2_CO_3_) is produced as an intermediate product during the second stage of decomposition, which occurs between 200 and 300 °C, and is eliminated during the third stage of decomposition, which occurred between 600 and 700 °C^33^. The first weight loss in the case of the SA/ZSM-5 composite was detected at a range from 40 to 94 °C and was related to the release of absorbed water molecules and the loss of volatiles. Between 94 and 150 C, there was a major weight loss due to the thermal degradation of the SA carbon chains and the production of Na_2_CO_3_^34^. The thermal stability of the composites is increased by blending ZSM-5 with alginate. Alginate's COO groups may interact with clay's OH groups to produce hydrogen bonds that are difficult to crack and increase the heat stability of the composites. This explains why composites exhibit less thermal deterioration than SA^33^. Dispersed ZSM-5 particles might also serve as a thermal barrier. TGA analysis thus indicates the SA/ZSM-5 nano composite's stability below 200 °C, whereas SA on the surface degraded above this temperature^35^.

#### Fourier transform infrared (FTIR)

**Figure 5:**
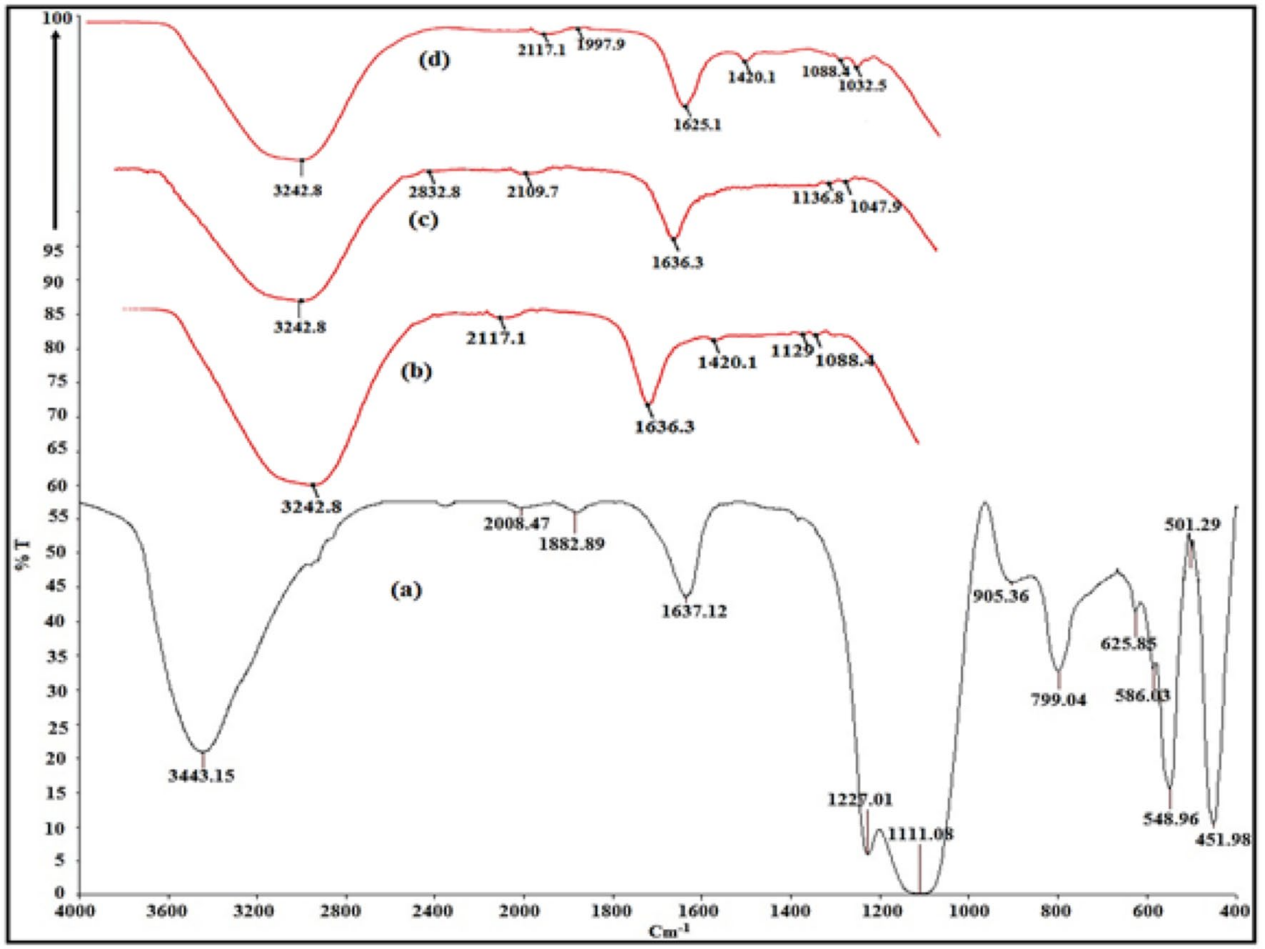
FTIR spectrum of ZSM-5 (**a**), SA (**b**), SA/ZSM-5 (**c**), and SA/ZSM-5 loaded with Ni (II) (**d**).

The Fourier Transform Infrared (FTIR) spectroscopy of ZSM-5, SA, SA/ZSM-5, and SA/ZSM-5 loaded with Ni (II) were meticulously examined to comprehend the underlying mechanism in the adsorption process. The FTIR spectra of ZSM-5 revealed a wide band ranging from 3350 to 3650 cm^−1^, corresponding to the hydroxyl group's stretching vibration (Fig. 5a). This is attributed to Si–OH and Al–OH groups present on the ZSM-5 surface and ^−^OH groups from adsorbed water. The interaction of hydrogen bonds between ammonium and silanol leads to an intensified vibration of the silanol group on the surface. Furthermore, the FTIR spectra showcased Si–O bond absorption peaks at 1111, 799, and 548 cm^−1^, indicating transverse asymmetric strain, asymmetrical strain vibrations, and buckling vibrations of Si–O–Si bonds: a distinct characteristic of ZSM-5 (Fig. 5a). Similar results on a typical ZSM–5 structure reported that zeolite lattice vibrations at 1100 and 540 cm^−1^ were attributed to the insensitive internal tetrahedron asymmetric stretching and bending vibration^36^. Both structure-sensitive exterior tetrahedron and structure-insensitive internal tetrahedron symmetric stretching vibrations are relevant for the band at about 799 cm^-1^. The double-ring tetrahedral vibration and asymmetric stretching of Si and AlO4 tetrahedra in the ZSM-5 framework are exhibited by peaks at 548 cm^−1^ and 1227 cm^−1^, respectively^37^. The FTIR spectrum of SA is depicted in Fig. 5b, with the O–H bond stretching vibration of the SA polymer occurring between 3000 and 3600 cm^−1^^38^. The symmetric and asymmetric stretching vibrations of the carboxylate group correspond to the band located at 1420 cm^−1^. Vibrational modes associated with the pyranose ring's C–O ring and C–O stretching, along with contributions from C–C–H and C–O–H deformation, are pertinent for bands at 1088 cm^−1^ and 1129 cm^−1^^39^. As hydroxyl groups arise at 3242 cm^−1^ and weaken the O–H bond during hydrogen bonding events, overlaying SA onto ZSM-5 spectra results in a shift of SA's vibrational modes^40^. In Fig. 5c, compared to pure alginate, the stretching vibration frequencies within carboxyl groups are situated at 1636 and 1420 cm^−1^. The identified high wave number shifts indicate that hydrogen bonding occurs between hydroxyl groups of ZSM-5 and alginate's OH and COOH groups. Strong, distinct absorption peaks around 3443, 3242, and 3242 cm^−1^ were observed across all spectra, relating to external water overlap and potential hydrogen bonds (^−^OH) within ZSM-5, alginate, and ZSM-5/alginate. Characteristic peaks of –COO^−^ stretching vibrations, ^−^OH groups, Si–O, and Al–O were also discerned in the SA/ZSM-5 composite spectra. This evidence substantiates the successful loading of SA into ZSM-5. Furthermore, the composite did not introduce any novel groups, verifying that this alteration was purely a physical process.

The ZSM-5, SA, and SA/ZSM-5 composite FTIR spectra displayed a peak at 1636 cm^−1^ before adsorption due to carboxylic groups, and then shifted to 1625 cm^−1^ (SA/ZSM-5 with Ni), indicating that Ni (II) adsorption was included (Fig. 5d). The ZSM-5 showed the presence of silanol bonds at absorption peaks at 1111, 799, 625, and 548 cm^−1^ owing to the existence of transverse asymmetric strain of Si–O–Si, symmetric stretching vibrations of Si–O–(Si), vibrations of Si–O–Al and buckling vibrations of bonds Si–O–Si respectively which are disappeared due to coating by SA and adsorption of Ni (II). Accordingly, the absence of silanol group in the FTIR spectrum could be because of reaction with Ni (II) or SA coating. The presence of silanol bonds is denoted in ZSM-5 through absorption peaks at 1111, 799, 625, and 548 cm^−1^ as a result of transversal asymmetric strain of Si–O^41^.

### Adsorption studies

#### Equilibrium time determination

**Figure 6:**
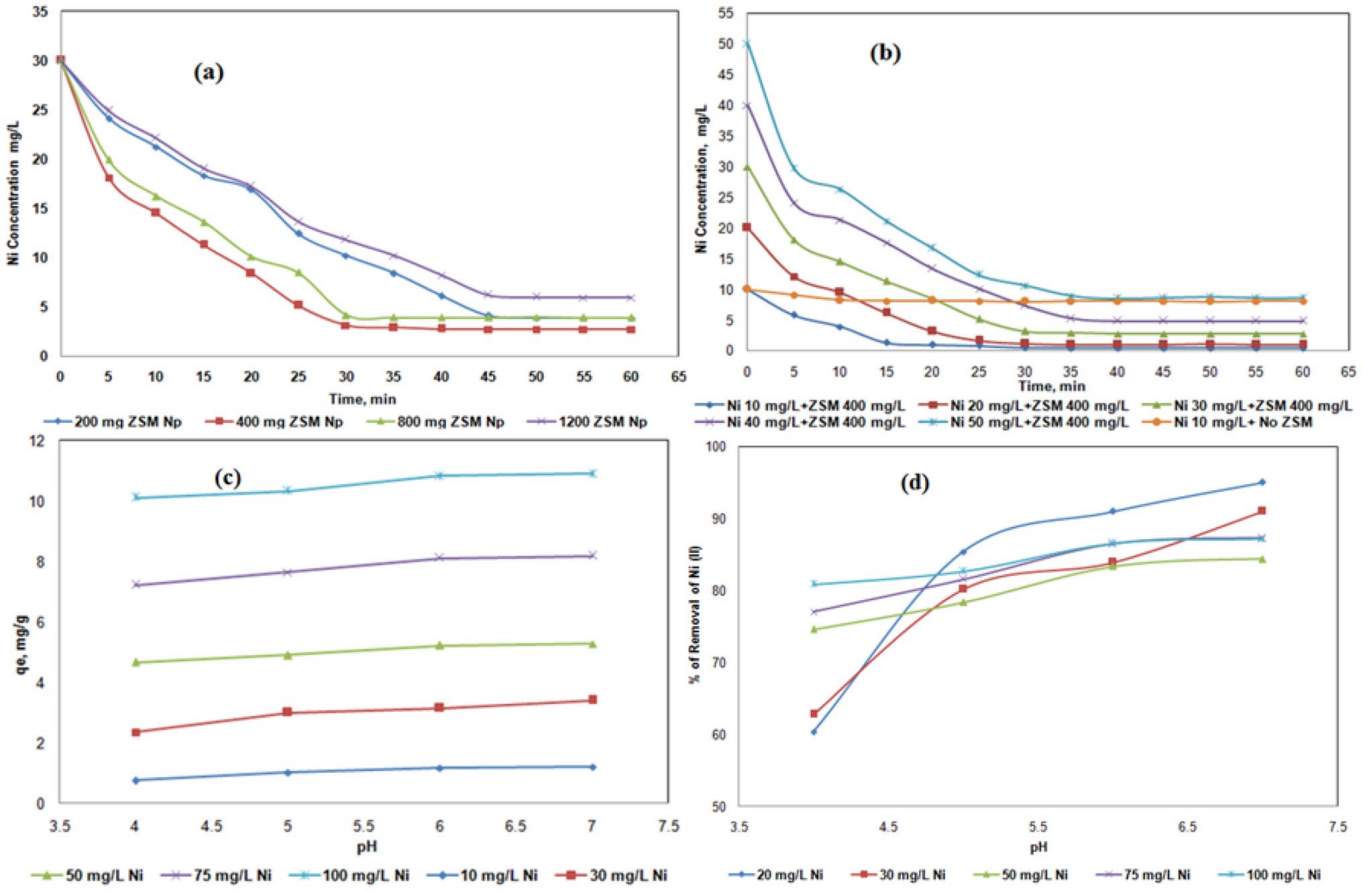
Effect of time on the adsorption Ni (II) with various quantities of SA/ZSM-5 beads (**a**), various Ni (II) concentrations with a fixed weight of SA/ZSM-5 beads (**b**). Effect of pH on sorption of Ni (II) uptake (**c**) and % of Ni (II) removal (**d**) using SA/ZSM-5 composite.

During the investigation, initial tests involving 50 mL of Ni (II) solution with varying concentrations of SA/ZSM-5 (200, 400, 800, and 1200 mg L^−1^) were performed to establish the required equilibrium time. The percent adsorption of Ni (II) at concentrations of 10, 20, 30, 40, 50, 75, and 100 mg L^−1^ was assessed using SA/ZSM-5 beads at a concentration of 400 mg L^−1^, adjusted to pH 6 and agitated at a speed of 160 rpm for durations ranging from 5 to 60 min. Within the first 25 min, nickel removal was found to surpass 50%, with the removal rate diminishing steadily until reaching the optimal contact period of 45 min as demonstrated in Fig. 6a. At this point, total nickel absorption had reached approximately 80%, with no discernible change afterward.

Subsequent experimentation dictated that the ideal contact time was set at 45 min for ensuing sorption studies. A decline in adsorption capacity was observed at the highest sorbent dose (1200 mg SA/ZSM-5), possibly attributable to an escalating amount of adsorbent constricting unoccupied adsorbent sites and thereby reducing such sites per unit mass. As a result, lesser Ni (II) ions were adsorbed in this case. Additionally, overlapping particles within the beads induced by an excessive amount of adsorbent decreased the overall surface area while simultaneously increasing the diffusion path. Figure 6b exhibits data concerning Ni (II) ion concentration in solution over time. A swift uptake was observed during initial stages of contact; however, it decelerated upon approaching equilibrium (Fig. 6b). The availability of numerous unoccupied active sites could explain the rapid uptake of Ni (II) ions initially, whereas their subsequent occupation may have contributed to the process slowing down^42^. Moreover, diffusion resistance could be another factor responsible for the deceleration of this phenomenon. Such observations correspond with previous studies on biomass in SA beads and molecular sieves for Ni (II) removal^43,44^.

#### Effect of pH

The pH of the adsorption medium is a key factor that influences the adsorption capacity^45^. pH is directly correlated with the capacity of hydrogen ions and metal ions to bind to active sites on the sorbent surface^46^. When the pH was raised from 4 to 6, the percentage of Ni (II) removal was improved from 74.5 to 84.4% on SA/ZSM-5 beads at the initial Ni (II) concentration of 50 mg L^−1^ (Fig. 6c). At pH 6, the maximum adsorption was observed to be 86% and, as the pH increased, Ni (II) sorption quickly decreased. Ni^2+^, Ni(OH)_3_, Ni(OH), and Ni(OH)_4_^–2^ are four nickel species that depend on the pH of an aqueous solution. Ni(OH)_2_ is formed at higher pH levels, which is the main reason for the decrease of the uptake by sorption. The formation of Ni(OH)_2_ at higher pH values were reported in the literature on Jordan Natural Zeolite^42^, clay^47^ and chemically modified sunflower biomass^18^. The percentage of Ni(II) ions removal ranged between 86 and 87% on 100 mg L^−1^ of Ni (II) solution at pH 6 and 7 respectively (Fig. 6d). The optimal pH was considered as pH 6 for Ni (II) on SA/ZSM-5 beads.

#### Effect of initial metal ion concentration

**Figure 7:**
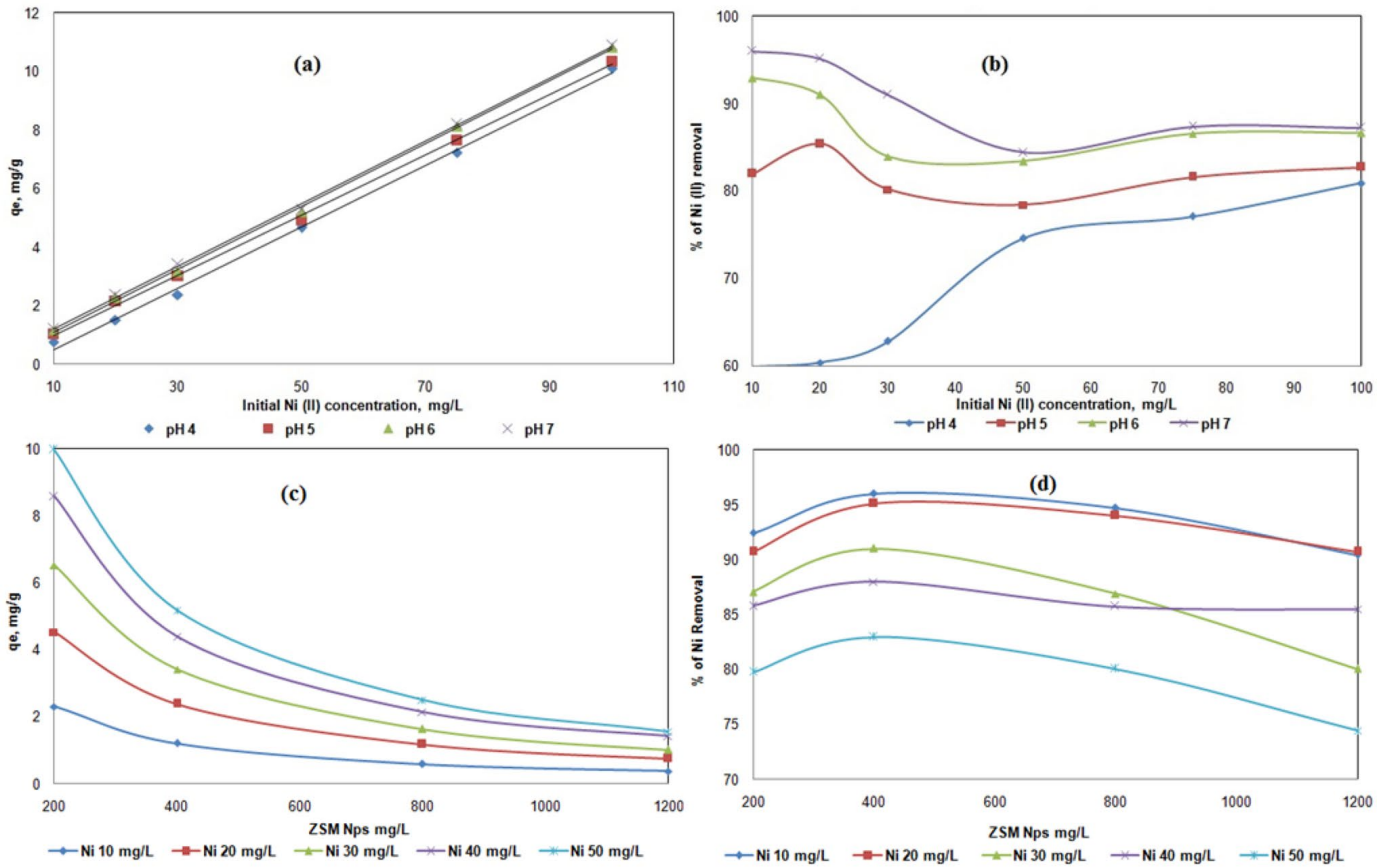
Effect of initial Ni (II) on sorption of Ni (II) uptake (**a**) and % of Ni (II) removal (**b**); effect of composite dosage on sorption of Ni (II) uptake (**c**) and % of Ni (II) removal (**d**) using SA/ZSM-5 composite.

Increasing Ni (II) initial concentration from 20 to 100 mg L^−1^, led to an increase of the biomass's adsorption capacity (q_e_) from 2.275 to 10.825 mg g^−1^ (Fig. 7a). The increase in initial metal concentration from 20 to 100 mg L^-1^ would also, decrease the removal of metal uptake from 93 to 86% at temperature 25 °C, pH 6 and a sorbent dosage of 8 g L^−1^ (Fig. 7b). This can be attributed to the above discussed factors, as extra sorption sites on SA/ZSM-5 were revealed to be rare. The mass transfer restriction between the solid phase and the aqueous phase can be successfully improved by increasing the initial Ni (II) concentration, leading to more metal ion adsorption. The similar trend was observed with adsorbents natural zeolite^48^ and copper oxide nanoparticles^19^ for the removal of Ni (II). Therefore, it may be concluded that the initial metal concentration is a driving force for the sorption process, as supported by earlier research^48^.

#### Effect of sorbent dosage

The potential of sorbent was examined by varying amounts of SA/ZSM-5 at pH 6 at 25 °C and various initial Ni (II) concentration solutions (20–100 mg L^−1^). The number of binding sites to remove metal ions at a given initial metal ion concentration increases when the sorbent weight is increased. When the SA/ZSM-5 concentration was increased to 4, 8, 16, and 24 g L^−1^, the Ni (II) uptake was reduced. At an initial Ni (II) concentration of 50 mg L^−1^, the adsorption capacity was found to be 9.9, 5.1, 2.5, and 1.5 mg g^−1^, respectively (Fig. 7c). When the sorbent dosage was changed from 4 to 8 g L^−1^ at an initial concentration of 50 mg L^−1^, the percentage of Ni (II) removal on SA/ZSM-5 increased from 79 to 83% at a pH value of 5.5 (Fig. 7d). The percentage of clearance decreased by 80% and 74%, respectively, when the sorbent dosage was further raised to 4 and 24 g L^−1^ with all sorbent weights, the same trend was observed in fixed initial Ni (II) concentrations. Two variables may be responsible for this, (i) A significant amount of adsorbent efficiently minimizes the degree of unsaturation of the adsorbent sites, hence minimizing the number of such sites per unit mass. As a result, less Ni (II) is adsorbed, and (ii) Particles overlap in the beads due to a large amount of adsorbent reducing the total surface area and extending the diffusion path. These two factors caused lowering adsorption per unit mass^49^.

#### Effect of temperature

**Figure 8:**
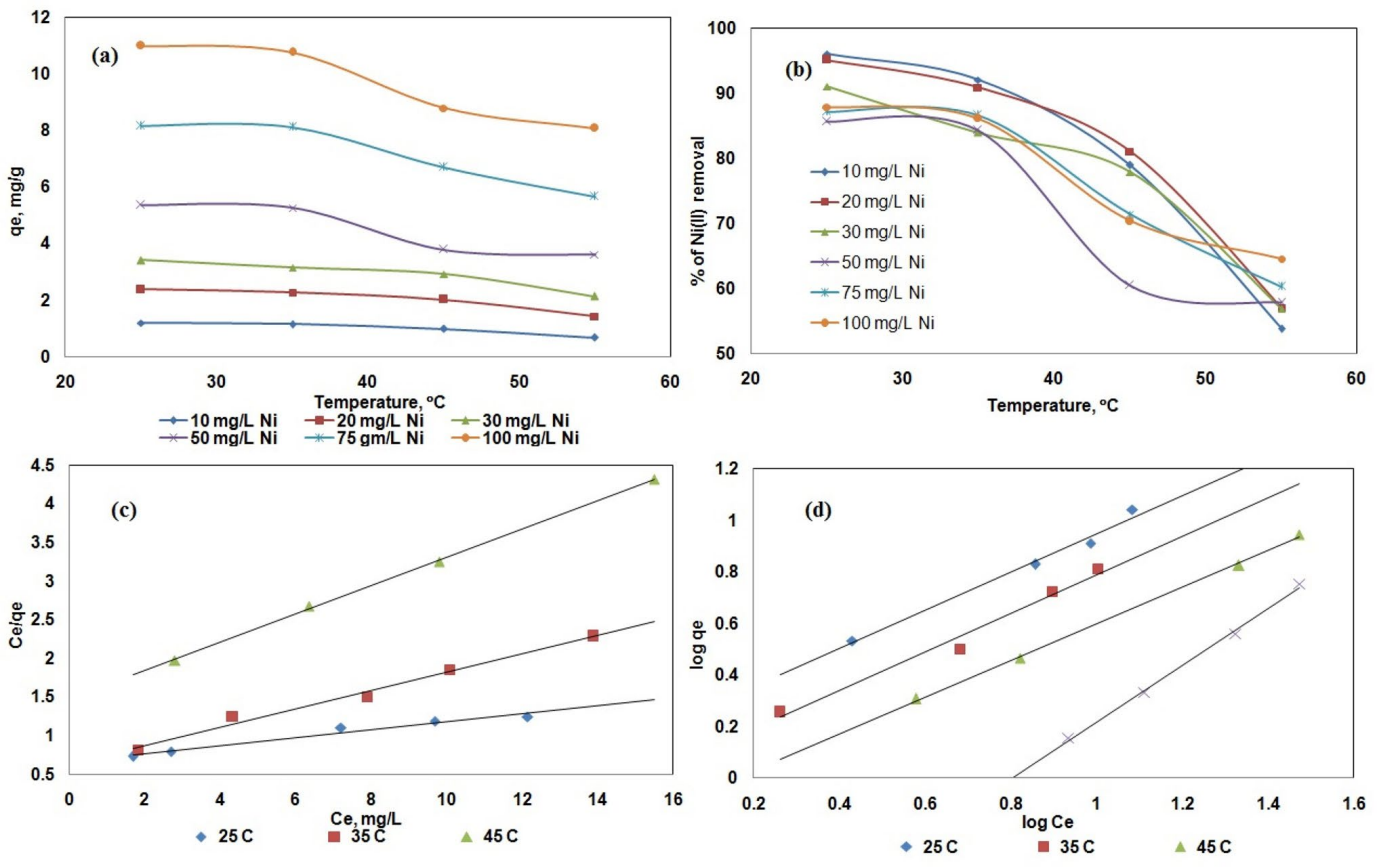
Effect of temperature on Ni (II) uptake (**a**), % of Ni (II) removal (**b**), Langmuir plot (**c**), Freundlich plot (**d**) on SA/ZSM-5 composite.

In the temperature range of 25–55 °C, the influence of temperature on the adsorption efficiency of SA/ZSM-5 for the removal of Ni (II) was studied. Ni (II) uptake decreased from 5.35 to 3.62 and 10.98 to 8.06 mg g^−1^ for initial concentrations of 50 and 100 mg L^−1^ under optimal pH 6 conditions at 8 g L^−1^ SA/ZSM-5 (Fig. 8a) with the increase in temperature from 25 to 55 °C. Similar effects were shown in all initial concentrations of Ni (II) (20, 30, 75, and 100 mg L^−1^). The % removal of Ni (II) showed by SA/ZSM-5 decreased from 85 to 57% and 87 to 64% with increasing the temperature range of 25–55 °C at Ni (II) concentration of 50 and 100 mg L^−1^ respectively (Fig. 8b). It might be because a temperature rise tends to cause the adsorbed metal ions at the interface with the solution to desorb. Because temperature variations can affect chemical adsorption because of changes in a molecule's kinetic energy, it is obvious that the removal rate increases as the temperature increases. In this event, Ni (II) was forced to gain more kinetic energy due to the increase in temperature to diffuse into the solid phase of the SA/ZSM-5 from 25 to 35 °C more quickly. Later further temperature increases found uptake capacity and percent removal decreased due to may be increased solubility of nickel hydroxide. Further, the reaction between SA/ZSM-5 and Ni (II) is exothermic due to lowered surface activity. The same results were observed on Jordan natural zeolite^42^, plant leaves^50^ marine bacteria^51^, and marine algae^52^.

### Adsorption isotherm models

The acquired data represented by sorption isotherms can be used to develop a relationship between the amount of Ni (II) sorbed per unit of SA/ZSM-5 (qe) and the Ni (II) concentration in the solution at equilibrium (C_e_). The sorption isotherms were investigated using two Langmuir and Freundlich equilibrium models.

#### Langmuir isotherm

The Langmuir theory's fundamental premise is that sorption occurs at homogenous sites within the sorbent. This model can be expressed by Eq. (1), which can be written in a non-linear form^53^.

1 $$\frac{{C_{e} }}{{q_{e} }} = \frac{1}{{q_{m} b}} + \frac{{C_{e} }}{{q_{m} }}$$

where b is a Langmuir constant for the affinity of binding sites (L mg^−1^) and *q*_*m*_ is the maximum adsorption capacity of Ni (II) per unit weight of SA/ZSM-5 at *C*_*e*_ (mg g^−1^). The slope (1/qm) and the intercept (1/b) can be calculated from the plots between (C_e_/q_e_) and C_e_ that are shown in Fig. 8c. According to the isotherms at varying temperatures, the SA/ZSM-5's maximum capacity (q_m_) and Langmuir adsorption affinity constant (b) were estimated. The correlation coefficients are shown in Table 1. The maximal capacity (q_m_) and sorption affinity constant (b) were 19.60 mg g^−1^ and 0.034 L mg^−1^, respectively, with an R^2^ of 0.964, indicating that the Langmuir model could best explain the current sorption data.

**Table 1:** Langmuir isotherm model parameters and R_L_ values at different initial metal ion concentrations for Ni (II) sorption by ZSM-5-alginate.

Temp (^°^C)	Langmuir constants			Freundlich constants			Separation factor	
	q_m_ (mg g^−1^)	b (L mg^−1^)	r^2^	K_F_	n_f_	r^2^	C_0_ (mg L^−1^)	R_L_
25	19.60	0.034	0.964	0.625	1.345	0.986	100	0.2269
35	8.470	0.074	0.986	0.914	1.341	0.981	100	0.1177
45	5.494	0.269	0.999	1.294	1.408	0.999	100	0.0357

The stronger electrostatic force of attraction was shown by the higher adsorption capacity, q_m_. A dimensionless parameter RL was calculated from the value of b and noted between 0 and 1 at various temperatures. Then, as the temperature increased, these values decreased, indicating that the process was desirable (Table 1). For the absorption of Ni (II) with other sorbents, a comparison of maximum metal absorption (q_m_) was shown in Table 2.

**Table 2:** Comparison of maximum metal uptake (q_m_) for sorption of nickel with other sorbents.

Biosorbent	q_m_ (mg g^−1^)	References
β-zeolite	10.19	^54^
β-zeolite-ethylenediamine derivative	12.22	^54^
Zeolite 3A	16.43	^55^
Clinoptilolite	13.1	^56^
Modified zeolite	25.41	^55^
*Pinus sylvestris* sawdust	15.68	^57^
Copper oxide (CuO) nanoparticles	15.4	^19^
Rice husk	16.7	^58^
SA/ZSM-5	19.60	Present study

#### Freundlich isotherm

Boedecker first presented the Freundlich adsorption isotherm, which Freundlich later modified^59^. The following is an example of the Freundlich adsorption Eqs. (2 and 3):

2 $$q_{e} = K_{f} C_{e}^{{\frac{1}{{n_{f} }}}}$$

Taking logarithms on both sides,

3 $${\text{ln}}q_{e} = {\text{ ln}}K_{f} + \frac{1}{{n_{f} }}{\text{ln}}C_{e}$$

where "qe" stands for equilibrium adsorption capacity (mg g^-1^), "Ce" for equilibrium adsorbate concentration in solution, "K_f_" for constants linked to the adsorption process, such as adsorption capacity and "n_f_" for adsorption intensity, and "qe" for equilibrium adsorption capacity.

The isotherm developed for the sorption of Ni (II) onto SA/ZSM-5 and fitted successfully at different temperatures is shown linearly in the plots in Fig. 8d. At 25 °C, n_f_ and K_f_ are determined to be 0.625 and 1.345 respectively, 0.914 and 1.341 respectively, and 1.294 and 1.408 respectively at 45 °C (Table. 1). Elevated correlation coefficient values indicate the efficacy of the Freundlich model in accurately representing sorption data. The Freundlich constant (n_f_) within the range of 1 to 10, exhibits a propensity towards SA/ZSM-5-facilitated sorption. This implies that the metal ion under investigation can be effectively sequestered from its aqueous solution via robust adsorption processes.

### Thermodynamic parameters

The Gibbs free energy ΔG is the basic criterion for determining whether a chemical process will occur. You can also use the sign and magnitude of the change in free energy ΔG to assess the spontaneity of the response. A negative sign of ΔG indicates the unpredictability of the chemical process. To design a chemical process system, one must first understand the changes that are expected to occur in a chemical reaction. In process device design, rate and degree of change carry more information. Considering the above, an analysis was performed on the influence of thermodynamic parameters on Ni (II) adsorption on SA/ZSM-5 particles. Thermodynamic parameters such as a change in standard free energy change ΔG°, Enthalpy ΔH°, Entropy ΔS° for any given adsorption process could be determined from the Eq. (4):

4 $$\Delta G^{0} = - {\text{RTlnKc}}$$

where ΔG° is the free energy change, expressed as J mol^−1^. b is the Langmuir equilibrium constant, The values of b (Table 1) at specific temperatures have been processed consistent with the following Van’t Hoff Eq. (5)^60^.

5 $${\text{lnb }} = - \frac{{\Delta {\text{H}}^{{0}} }}{{{\text{RT}}}}{ + }\frac{{\Delta {\text{S}}^{{0}} }}{{\text{R}}}$$

**Figure 9:**
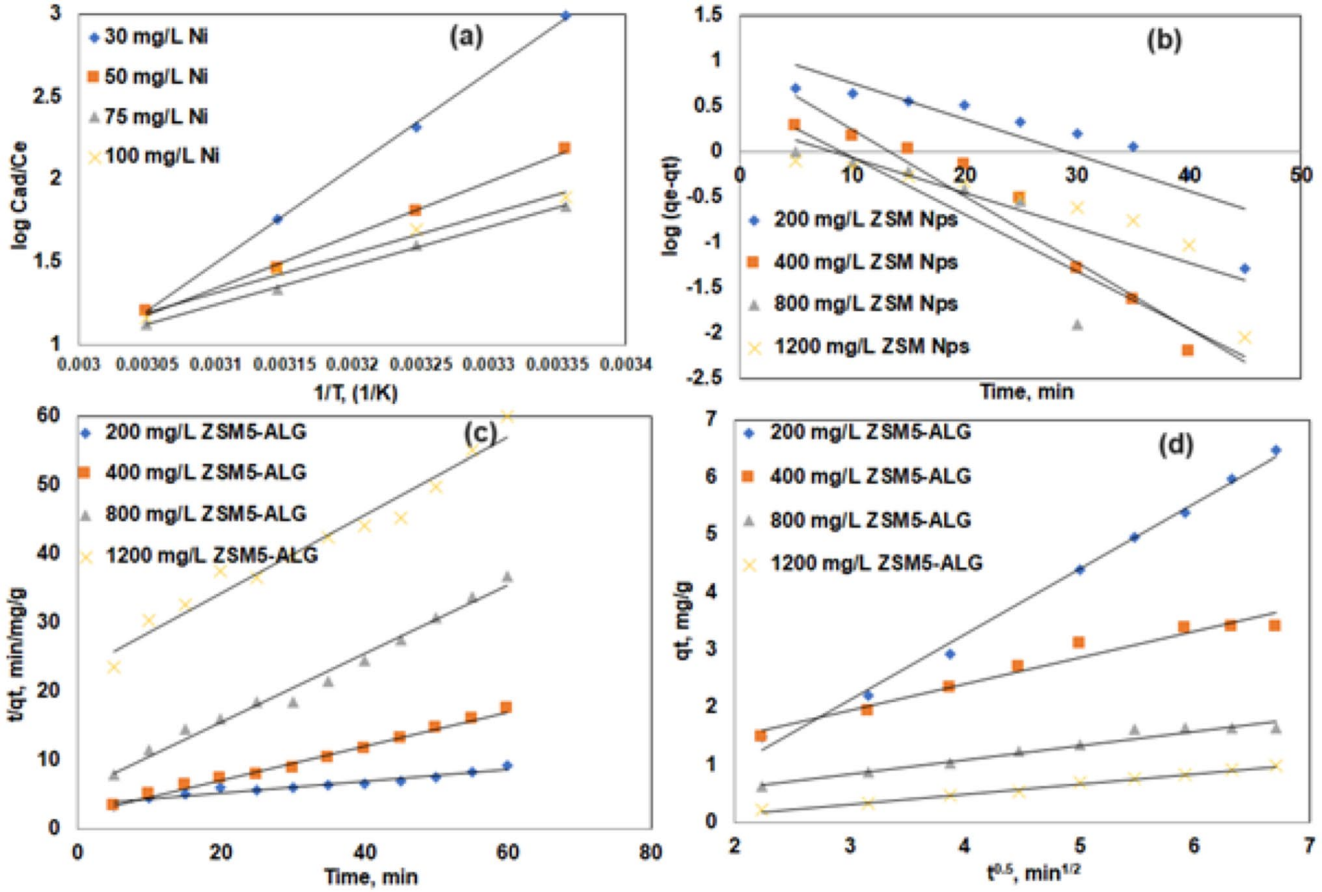
Van’t Hoff plot on sorption of Ni (II) (**a**), first-order kinetics (**b**), pseudo-second order kinetics (**c**), intraparticle diffusion (**d**) on SA/ZSM-5.

R is the universal gas constant, and b is in L mol^−1^ (8.314 J mol^−1^ K). The plot of ln b plotted versus 1/T, which is shown in Fig. 9a, was used to determine the enthalpy changes (H) and entropy changes (S) for the adsorption process of Ni (II) on SA/ZSM-5 beads. Table 3 displays the calculated thermodynamic data.

**Table 3:** Thermodynamic parameters for Ni (II) sorption on SA/ZSM-5.

C_0_ (mg L^−1^)	$\Delta H$^0^ (KJ mol^−1^)	$\Delta S$^0^ (J mol^−1^ K)	$\Delta G$^0^ (KJ mol^−1^)	Temp. (^°^C)
30	−110.498	−136.266	−40.496	25
50	−61.615	−71.75	−22.039	35
75	−45.225	−50.549	−16.240	45
100	−45.034	−49.651	–	–

The spontaneous nature of the sorption process is shown by a large negative value for G°. For the adsorption of Ni (II), the free energy values increased positively as the temperature increased, indicating that the spontaneity of the sorption process decreases as temperature rises. The exothermic character of the adsorption was shown by the negative H° readings. The negative values of S° demonstrated a reduction in the randomness at the solid/solution interface during the adsorption of Ni (II) ions on leaf biomass^50^. The same behavior was observed on, natural bentonite^61^, TiO_2_-alum sludge^62^, and β‑zeolite^63^.

### Adsorption kinetic models

The kinetic data is used to determine the optimum processing conditions for a large-scale batch or continuous operation and helps to trace the rate-determining step of the transport mechanism. This study used pseudo-first order and pseudo-second-order kinetic models to attempt to fit the available sorption data.

#### Pseudo-first-order/Lagergren kinetic model

The pseudo-first-order or Lagergren kinetic rate equation for liquid–solid adsorption was developed based on the capacity of solids for adsorption. For solute adsorption from a liquid solution, it is one of the most popular adsorption rate equations^64^.

Written as follows is the pseudo-first-order kinetic Eq. (6):

6 $$\tfrac{{dq_{{}} }}{dt} = k_{1} \left( {q_{e} - q_{t} } \right)$$

where qt is the quantity of solute adsorbed at any given time 't', qe is the amount of solute adsorbed at equilibrium per unit mass of adsorbent (mg g^-1^), and k_1_ is the rate constant. Equation (7) is produced by applying the boundary conditions and simplifying.

7 $$\ln (q_{e} - q_{t} ) = \ln q_{e} - k_{1} t$$

The slope of the linear plot between ln(q_e_ − q_t_) vs. 't' for various adsorption parameters, including pH, temperature, adsorbate concentration, adsorbent dose, and particle size, can be used to calculate 'k_1_'. At a given temperature and pH, a first-order kinetic plot for Ni (II) is drawn using SA/ZSM-5. The pseudo-first-order rate constant k1 could be determined by measuring the slope of the plot between log (qe − qt) and time, t. The adsorption of Ni (II) using SA/ZSM-5 does not obey the Lagergren pseudo-first-order kinetic plot well since it does not follow a straight line, as seen in Fig. 9b.

#### Pseudo-second-order kinetic model

A pseudo-second-order reaction model was used to evaluate the fitness of the sorption data considering the data. According to^65^, the rate expression for the pseudo-second-order reaction model is as follows in Eq. (8):

8 $$\tfrac{dq}{{dt}} = k_{2} (q_{e} - q_{t} )^{2}$$

Equation (9) becomes, with integration for boundary conditions when t = 0 to t > 0 and q_t_ = 0 to q_t_ > 0 and further simplifications.

9 $$\tfrac{t}{{q_{t} }} = \tfrac{1}{{k_{2} q_{e}^{2} }} + \tfrac{1}{{q_{e} }}t$$

The values for q_e_ and k_2_ were determined from the linear relationship shown by the plot of t/q_t_ vs t of Eq. (10) (Fig. 9c). The pseudo-second-order kinetic model's rate constants and correlation coefficients are listed in Tables 4 for reference. These findings show that the nickel metal adsorption data for both biosorbents were well-fit by the pseudo-second-order kinetic plots. It can predict the kinetics of Ni (II) adsorption on SA/ZSM-5 using this kinetic model.

**Table 4:** Kinetic parameters for Ni (II) sorption on SA/ZSM-5.

Biomass weight in matrices, g L^−1^	Pseudo-first-order	Pseudo-second-order	Intra-particle diffusion
4	*qe_exp_ = 13.215 mg g^−1^		
	*qe_cal_ = 14.288 mg g^−1^	qe_cal_ = 11.6 mg g^−1^	k_p_ = 1.142 mg h^0.5^ g^−1^
	k_1_ = 0.0898 h^−1^	k_1_ = 2.08 × 10^−4^g/(mg h)	I = 1.304
	R^2^ = 0.768	R^2^ = 0.962	R^2^ = 0.994
8	qe_exp_ = 8.572 mg g^−1^		
	qe_cal_ = 9.616 mg g^−1^	qe_cal_ = 4.048 mg g^−1^	k_p_ = 0.459 mg h^0.5^ g^−1^
	k_1_ = 0.1681 h^−1^	k_1_ = 2.78 × 10^–3^ g/(mg h)	I = 0.557
	R^2^ = 0.928	R^2^ = 0.990	R^2^ = 0.959
16	qe_exp_ = 5.35 mg g^−1^		
	qe_cal_ = 5.616 mg g^−1^	qe_cal_ = 2.020 mg g^−1^	k_p_ = 0.247 mg h^0.5^ g^−1^
	k_1_ = 0.142 h^−1^	k_1_ = 4.25 × 10^–3^ g/(mg h)	I = 0.105
	R^2^ = 0.708	R^2^ = 0.984	R^2^ = 0.954

*qe_*exp*_ experimental qe value, *qe*_*cal*_ calculated qe value.

#### Intraparticle diffusion model

Film or external surface diffusion, pore diffusion, surface diffusion, pore surface adsorption, or a combination of one or more processes can all be used to affect adsorption. An apparent diffusion coefficient that corresponds to experimental data on adsorption rates can be used to relate the diffusive mass transfer in a quickly agitated batch operation. If the rate at which the components diffuse toward one another determines the rate of a process, such a process is said to be diffusion controlled. The formal form of Weber's diffusion model is represented by Eq. (10).

10 $$q_{t} = k_{ip} \times t^{\frac{1}{2}} + I$$

where k_ip_ (mg g^−1^ min^−1^) is the intraparticle diffusion rate constant and q (mg g^−1^) is the concentration of adsorbate in the solid phase at time t (min). The intercept displays the thickness of the boundary layer on the adsorbent surface as a linear plot of q_t_ versus t. (Fig. 9d). The effect of the boundary layer was shown to be stronger the higher the value of I. Table 4 shows the results, with kip values of 1.142, 0.459, and 0.247 mg h^0.5^ g^−1^, and boundary thickness 'I' values of 1.304, 0.557, and 0.107 for 4, 8, and 16 g L^−1^ SA/ZSM-5. The border thickness decreases with increasing sorbent weight, proving that intraparticle diffusion is not the adsorption process' rate-limiting stage.

## Materials and methods

### Synthesis of ZSM-5

The materials used in this work for the synthesis were aluminum sulfate, (Al_2_(SO_4_)_3_.18H_2_O, laboratory-grade, Merck), silica gel (grade 923, 100–200 mesh, Aldrich), tetra propylammonium bromide (C_12_H_28_BrN, Merck), concentrated sulfuric acid (H_2_SO_4_, 98%, Aldrich), sodium hydroxide (flake, Merck) and ammonium nitrate (NH_4_NO_3_, BDH). Zeolite synthesis was carried out in a 450 cm^3^ high-pressure stainless steel autoclave reactor built by Parr Instrument Company (Parr model 4562), USA.

The following process was used to make ZSM-5 from a hydrogel combination including silicon, aluminum, and a template: Solution A (pH 1) was made by dissolving a specified amount of aluminum sulfate in deionized water and then adding the needed amount of sulfuric acid solution after full digestion. Solution B was made by dissolving silica gel powder in an alkaline solution at 100 °C, resulting in a sodium silicate solution with a composition of 29.50%wt SiO_2_, 10.50%wt. Na_2_O. Under vigorous agitation, solutions A and B were combined until a homogeneous gel mixture was produced. The gel combination was then added to the tetra propylammonium bromide solution, which was stirred for approximately 2 h. The pH of the final gel combination was 10.5. The gel mixture was then placed in the stainless-steel autoclave, where the synthesis took place for 24 h at 700 rpm at 180 °C. The solid product was filtered and rinsed multiple times with warm deionized water toward the end of the synthesis time. To eliminate the organic template trapped in the pores of zeolite, the solid result was dried at 120 °C and subsequently calcined at 550 °C for 3 h under airflow.

After calcination, Na-ZSM-5 was ion exchanged for 24 h at 80 °C under reflux with a 2M solution of NH_4_NO_3_ and a solution ratio of zeolite to solution of 1 g zeolite/10 cm^3^ solution. The NH^4+^/ZSM-5 was filtered and washed with deionized water after ion exchange, and the sample was calcined at 550 °C for 3 h under airflow to break down the ammonium ions into hydrogen form. For each sample, the ion exchange technique was performed twice^66^.

### Preparation of SA/ZSM-5 composite

The preparation of the SA solution was carried out using sodium alginate powder purchased from Loba chem-laboratory reagent, India (low viscosity: 1% w/w solution, 25 °C). 4% of the SA solution was prepared by dissolving 4 g of SA in 100 ml of distilled water and rapidly mixing to prevent lumps. 5% calcium chloride dihydrate (CaCl_2_⋅2H_2_O, Sigma Aldrich) was prepared with a concentration of 5% as a crosslinking agent. Nickel (II) chloride hexahydrate (NiCl_2_⋅6H_2_O, Sigma-Aldrich) was used to prepare the standard Ni (II) solution. Other chemicals for pH adjustment, such as HCL of analytical reagents, were utilized.

The ionotropic gelation method was used for the preparation of the SA/ZSM-5 nanocomposite. The aqueous solution of SA slowly dropped to a 5% CaCl_2_⋅2H_2_O crosslinking reagent (100 mL) with gentle stirring (200 rpm). The formed spherical beads were left overnight in CaCl_2_⋅2H_2_O solution to harden. The beads were then washed 5 times with distilled water, then dried and stored for investigation. A series of ZSM-5 nanoparticles (200, 400, 800, and 1200 mg L^−1^) nanoparticles incorporated in SA were prepared, mixed with SA, and strongly stirred using a hot plate stirrer at 40 °C. Then the prepared SA/ZSM-5 nanocomposite was dropped into 100 mL of 5% CaCl_2_⋅2H_2_O solution to allow the chemical binding of SA to the ZSM-5 surfaces.

### Sorption batch experiments

Batch adsorption experiments were carried out to study the kinetic parameters including reaction time, metal ion concentration, initial pH, sorbent dose, and temperature for the removal of Ni (II) from aqueous solution. The sorption study for each parameter was carried out in a 250-mL conical flask containing 100 mL of solution. The sorption time was carried out for a time from 0 to 120 min and the Ni (II) concentrations tested were 10, 20, 30, 50, 75, and 100 mg L^−1^. The dose of nanocomposite varied between 200, 400, 800, and 1200 mg L^−1^. pH study was carried out for the values of 4, 5, 6 and 7 using while the temperature was investigated at 25, 35, 45, and 55 °C.

For each experiment,100 mL of Ni (II) was brought into contact with SA/ZSM–5 nanocomposite in a conical flask and then, shaken uniformly by a wise shaker (model SHO-2D Dihane, Korea) at 200 rpm. After each experiment, the mixture is filtered from the nanocomposite and the residual concentration of nickel ion was determined before, and after treatment and was analyzed by ICP-OES according to^67^. The adsorbed amount of Ni ion on the developed nanocomposite adsorbent (q_e_) was calculated as shown in Eq. (11).

11 $$\mathrm{qe }(\mathrm{mg g}^{-1}) =\frac{(Ci-Cf)V}{m}$$

where q_e_ (mg g^−1^) is the number of pollutants adsorbed per unit weight of the adsorbent at equilibrium. C_i_ and C_f_ are the initial and final Ni (II) concentrations. While V is the volume of the aqueous solution and m (g) represents the weight of the nano adsorbent.

### Characterization of SA/ZSM-5 nanocomposite

Physical characterization and morphological structure of ZSM-5 and SA/ZSM-5 composites were carried out in terms of X-ray diffraction (XRD), thermogravimetric analysis (TGA), scanning electron microscopy (SEM) and Fourier transform infrared (FTIR). XRD was carried out by (PAN analytical, X'PRT PRO). Using Cu-target with Ni-filtered radiation (λ = 1.542 A°). The diffraction angle (2θ) ranged between 2° and 80°. The TGA was carried out using SETARAM Labsys TG-DSC16 equipment in the temperature range of ambient to 1000 °C under Argon flow. The SEM instrument used was a Jeol JSM 5300 from Japan, which had been coated with a thin gold film to make it conductive. Before analysis, all samples were placed on stubs and gold coated to make them electrically conductive. About 2500× magnification was used. FTIR measurement was performed using the Agilent Cary 630, in the wavelength range of 400–4000 cm^−1^.

## Conclusions

The study demonstrates that various parameters, including contact time, initial metal ion concentration, pH, temperature, and sorbent dosage, significantly influence the adsorption of nickel ions onto SA/ZSM-5 beads. The experimental data indicate an increase in Ni (II) ion removal efficiency with a rise in the initial metal concentration. However, the sorption performance diminishes at higher pH levels beyond 5.0 to 6.5. The study establishes that maintaining optimal conditions namely, sorbent dosage (8 g L^−1^), temperature (25 °C), and pH (6.5) can result in effective removal of Ni(II) ions from the solution. The results also highlight the adsorption capacity (q_e_) of SA/ZSM-5 beads as sensitive to both initial concentration and biomass dosage variations. Furthermore, the Ni (II) sorption capacity is negatively correlated with increasing temperatures when using an initial concentration of 100 mg L^−1^. The Langmuir and Freundlich equilibrium isotherm models are well-suited to fit the experimental findings, while the second-order kinetic model successfully depicts the adsorption kinetics of Ni (II) ions onto these beads. The thermodynamic characteristics of this process showcase negative values for free energy change, thereby confirming its feasibility and spontaneity. The exothermic nature is evident by the negative enthalpy values, whereas a decreased randomness at the solid–liquid interface is indicated by the negative entropy value.

## Data Availability

All authors ensure that all data and materials and software applications or custom code support their published claims and comply with domain standards. The datasets used or analyzed during the current study are available from the corresponding author on reasonable request.
